# Microbiota composition of dadih – a traditional fermented buffalo milk of West Sumatra

**DOI:** 10.1111/lam.13107

**Published:** 2019-01-31

**Authors:** K. Venema, I.S. Surono

**Affiliations:** ^1^ Centre for Healthy Eating & Food Innovation Maastricht University – Campus Venlo Venlo The Netherlands; ^2^ Food Technology Department Faculty of Engineering Bina Nusantara University Jakarta Indonesia

**Keywords:** back‐slopping, buffalo milk, dadih, fermentation, microbiota, pasteurization

## Abstract

Dadih is an Indonesian traditional spontaneously fermented buffalo milk, produced in West‐Sumatra, which is nutritious and has health benefits. The mechanism of action behind the health benefits is largely unknown, but several probiotic strains have been isolated from dadih, which may contribute to its health properties. To identify the composition of its microbiota, two artisanal dadih samples (*n* = 8) were collected from four producers. The raw buffalo milk used for fermentation was either pasteurized (*n* = 4) or not (*n* = 4), and back‐slopping was used as a starter‐culture (*n* = 5) or not (*n* = 3). DNA was extracted from each sample in duplicate and the microbiota composition was determined by 16S‐rRNA‐gene amplicon‐sequencing of the V3–V4 region. PCoA analysis showed clear separation of the samples by producer, but no separation due to pasteurization or use of back‐slopping. *Lactococcus* (52–83%) predominated in all samples, followed by *Klebsiella* (5–26%), and *Lactobacillaceae*,* Bifidobacterium* (particularly high (*c*. 18%) in the non‐pasteurized, back‐slopped product from Palupuh), *Streptococcus* and *Leuconostoc*. Back‐slopping practice correlated significantly with higher abundance of *Lactobacillaceae*,* Pediococcus*, species of the order *Burkholderiales*, and *Serratia*, but with lower abundance of several other Enterobacteriaceae (including *Klebsiella*), *Streptococcaceae*,* Staphylococcus* and *Brachybacterium*. Pasteurization was not significantly correlated with the presence of certain members of the final microbiota. Taken together, fermentation results differ significantly from producer to producer and back‐slopping practice would be advisable.

**Significance and Impact of the Study:**

Using state‐of‐the‐art methods we determined the microbiota composition of dadih, an artisanal, traditional fermented buffalo milk of West Sumatra with health benefits. We show that the artisanal practice leaves room for standardization and optimization with respect to the presence of potential pathogenic species in the final product. The Dadih Initiative in Indonesia aims to expand production of this health promoting product, and the findings help to determine important steps for potential food safety issues and good‐manufacturing‐practices to obtain a safe, nutritious and healthy traditional yoghurt‐like functional food.

## Graphical abstract

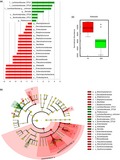

## Introduction

Dadih is a traditional fermented buffalo milk from West Sumatra, Indonesia, and has been used as food condiment by people in that region for a long time (Surono 2015a, 2016). In Indonesia, dadih is still a homemade product made in an artisanal manner, involving raw milk of the water buffalo. The raw buffalo milk is placed in short bamboo tubes, which are then covered with banana leaves, incubated at the ambient temperature (25–30°C) overnight, and allowed to ferment until it acquires a thick consistency (Akuzawa and Surono 2002; Surono 2015b). Various indigenous lactic acid bacteria (LAB) are involved in the dadih fermentation. Their composition in the final product may vary from time to time, as well as from one place to another, due to the natural, spontaneous fermentation practices used by different local producers (Surono and Hosono 2000; Akuzawa and Surono 2002). Dadih has been shown to have several health benefits, but mostly this is from anecdotal evidence or preclinical studies (Kusuma *et al*. 2015). However, the mechanism of action behind the health benefits of dadih is largely unknown, but several probiotic strains have been isolated from dadih, which may contribute to its health properties (Collado *et al*. 2007). *In vivo* in humans, *Lactobacillus plantarum* IS‐10506 or *Enterococcus faecium* IS‐27526 have demonstrated enhancement of humoral immune response, and nutrient absorption in several studies (Surono *et al*. 2011, 2014; Prakoeswa *et al*. 2017).

Only a few studies have been performed on the composition of the microbiota of the final dadih product (Hosono *et al*. 1989; Surono 2003; Syukur *et al*. 2014), but most have been focused on the potential health beneficial microbes present in the product. The process of dadih manufacturing as currently performed in an artisanal manner does not involve good hygiene practices. However, no incidence of product failure or food poisoning has been reported by people who consumed dadih (Surono 2015b). Given the interest in exploring the health beneficial properties of dadih further, the Dadih Initiative was initiated in Indonesia in 2017 to establish and standardize specifications for production and quality control of dadih, with the aim to produce large quantities of dadih for the local population. Before extensive market introduction, however, more insight into the microbial ecology and food safety of the product are needed. Recent developments in next generation sequencing now allow gaining more insight into the complexity of the microbial ecology of dadih, and through that the potential advanced applications of this understudied, invaluable resource for microbial bioprospecting, as well as a functional food.

Therefore, the aim of the study was to determine the microbiota composition of the final fermented product, studying various sites of collection, made from pasteurized or raw milk, and using back‐slopping or not.

## Results and discussion

Nucleic acids were extracted from duplicate aliquots of the collected dadih samples. For one of the samples (see Table 1), the quality of the DNA was insufficient to get high‐quality sequence data and that sample was removed from further analysis.

**Table 1:** Origin and characteristics of the collected dadih samples

	Site of collection	Pasteurized	Back slopping	Note
A	Gadut	Yes	Yes	Producer 1
B	Gadut	No	Yes	Producer 1
C	Palupuh	No	Yes	
D	Palupuh	Yes	No	
E	Padang Panjang	No	No	
F	Padang Panjang	Yes	No	
G	Gadut	Yes	Yes	Producer 2
H	Gadut	No	Yes	Producer 2

**Figure 1:**
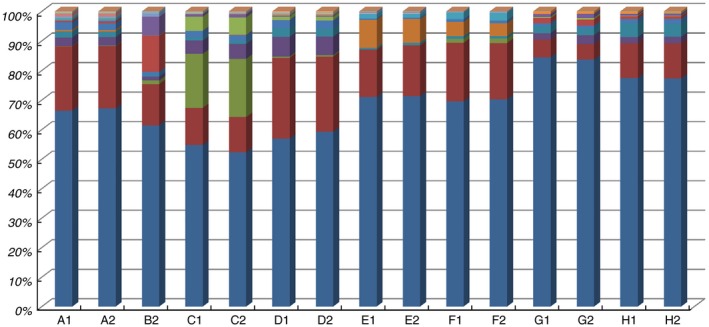
Relative abundance of major OTUs (at least 0·1% in any of the samples) in the duplicate dadih samples (coded according to Table 1). Legend for microbial OTUs (at genus level, or higher if not available at genus level, indicated by f__: family, o__: order. When the same names are given, these are still different OTUs): (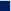) *Lactococcus*; (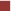) *Klebsiella*; (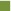) f_*Bifidobacteriaceae*; (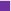) *Leuconostoc*; (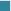) f__*Enterobacteriaceae*; (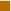) *Streptococcus*; (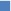) f__*Enterobacteriaceae*; (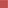) f__*Lactobacillaceae*; (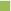) *Lactobacillus*; (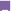) f__*Lactobacillaceae*; (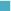) *Acinetobacter*; (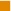) f__ *Oxalobacteraceae*; (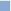) *Vagococcus*; (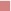) *Acetobacter*; (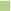) *Proteus*; (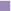) f__*Enterococcaceae*; (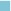) o__*Lactobacillales* and (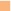) *Corynebacterium*.

*Lactococcus* (52–83%) predominated in all samples (Fig. 1; Table S1), followed by *Klebsiella* (5–26%), and *Lactobacillaceae* (0–18%; particularly high in the non‐pasteurized, back‐slopped product from Gadut, producer 1, sample B), *Bifidobacterium* (0–18%; particularly high in the non‐pasteurized, back‐slopped product from Palupuh, sample C), *Streptococcus* (0–9%; particularly in the Padang Panjang samples) and *Leuconostoc* (0–6%; particularly in the Palupuh samples). From Fig. 1, it is clear that the duplicate samples are very similar in composition, indicating the reproducibility of the sequencing method.

**Figure 2:**
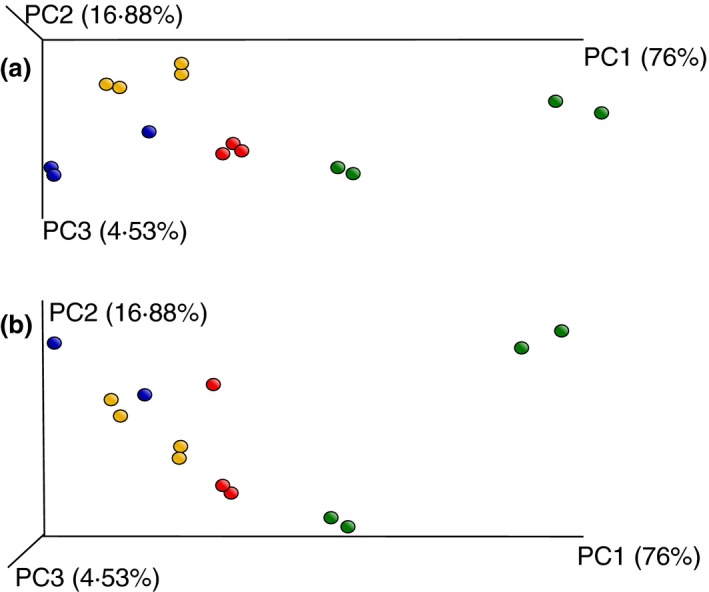
Weighted UniFrac of the dadih samples, colored according to producer (sampling site). a and b are two differently rotated representations of the same graph. (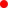) Gadut, producer 1; (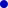) Gadut, producer 2; (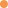) Padang Panjang and (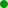) Palupuh.

**Figure 3:**
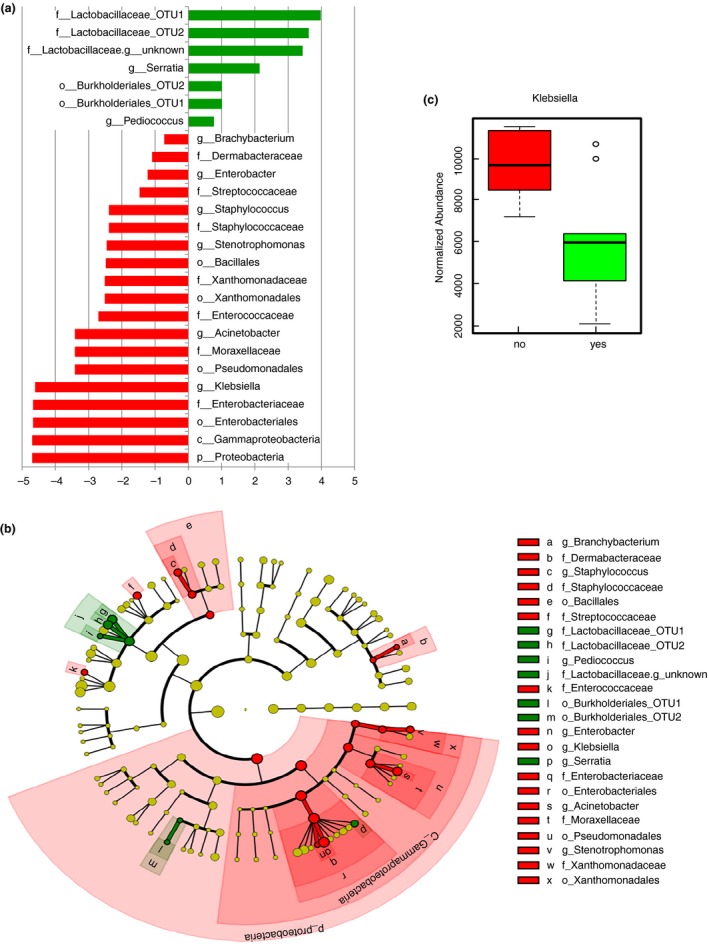
(a) Linear discriminant analysis effect size (LEfSe) on the dadih samples using back slopping (starter culture 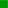) or not (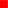). (b) Cladogram of the LEfSe result. (c) Difference in normalized *Klebsiella* abundance between samples using back‐slopping (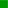) or not (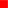), data obtained using metagenassist.

Principal coordinate analysis (PCoA; weighted UniFrac) using QIIME (Caporaso *et al*. 2010) showed clear separation of the samples by producer (Fig. 2), but no separation due to pasteurization or use of back‐slopping (Fig. S1). The separation by producer corroborates the artisanal production of the fermented buffalo milk, which is practiced slightly differently by each individual farmer, and at least results at different farms in different starter cultures and/or endogenous microbiota in the raw milk (Fig. 2). A field trip to some farmers indicated some practices that may be responsible for these local differences, including (i) cleaning of the tits with a (dirty) cloth, and (ii) allowing a baby buffalo to drink first to stimulate milk‐flow, before collecting the milk for dadih production. For large scale and safe production of dadih this clearly has to be standardized. Although PCoA did not show separation, using LEfSe (Segata *et al*. 2011), back‐slopping correlated significantly with higher abundance of *Lactobacillaceae*,* Pediococcus*, species of the order *Burkholderiales*, and *Serratia* (Fig. 3a,b), but with lower abundance of several other *Enterobacteriaceae* (including *Klebsiella*; Fig. 3c), *Streptococcaceae*,* Staphylococcus* and *Brachybacterium*. Pasteurization was not significantly correlated with the presence of certain members of the final microbiota (not shown).

This is when taking all samples from all producers and fermentation practices together. When looking at individual samples there are some differences observed. For instance, *Klebsiella* is high (>11% abundance) in all samples, except the pasteurized samples with back‐slopping from Gadut, producer 2 (5·5%). This is still rather high for a food product, but the combination of pasteurization and back‐slopping use apparently suppresses *Klebsiella* for this producer. This is not a general feature, as for Gadut producer 1, the abundance of *Klebsiella* in the pasteurized back‐slopped dadih is even higher (21%) than in the non‐pasteurized, back‐slopped product (13%). Apparently, pasteurization of the raw milk does not prevent growth of *Klebsiella* in the subsequent manufacturing process, suggesting *Klebsiella* is not necessarily present in the raw milk. *Bifidobacterium* is highest in the non‐pasteurized, back‐slopped product from Palupuh (18%), while it is virtually absent in the pasteurized product from the same producer (0·4%). Also in other products it is relatively low (0·09–1·4%), and even below the level of detection in the products from producer 2 from Gadut. *Leuconostoc* was absent (below the level of detection) in Padang Panjang samples, while it ranged from 1·3–3·8% in samples from both Gadut producers, and was 4·7% (non‐pasteurized, back‐slopped) to 6·3% (pasteurized, no back‐slopping) in the Palupuh products. In contrast, where *Leuconostoc* was absent in Padang Panjang samples, *Streptococcus* was the highest in these samples (4·5–8·5%). While in those samples high in *Leuconostoc*,* Streptococcus* was virtually absent (0·01% in 1 of the 4 samples from Palupuh; below level of detection in the other 3). These two genera seemed to be mutually exclusive, with the exception of the pasteurized, back‐slopped sample from Gadut, producer 1 where both were present, although Streptococcus at low abundance (0·6%). *Acetinobacter* showed a similar mutually exclusive presence with *Leuconostoc* as *Streptococcus*, also with low abundance in the pasteurized, back‐slopped sample from Gadut, producer 1. It is interesting to explore this mutual exclusivity further, as it might be based on antimicrobial activity. *Lactobacillacea* were absent in Padang Panjang samples, either pasteurized or not, and relatively high (18%) in the non‐pasteurized, back‐slopped sample from Gadut, producer 1. The only other samples with abundance >2% were the non‐pasteurized, back‐slopped samples from Palupuh (6%). Interestingly, in those samples that contained high *Klebsiella*, also high *Enterobacteriaceae* were present, with an almost linear correlation (Figs S2 and S3).

Figure S3 shows the correlation between the abundance of the different OTUs in the samples, created using the metagenassist pipeline (Arndt *et al*. 2012), indicating the co‐occurrence and mutual exclusion of certain microbes. Random Forest analysis (from within metagenassist; using generation of 500 trees, with 10 predictors to try for each node) showed that all 15 samples could be correctly classified as being fermented in the presence of a starter culture or not (Fig. S4 showing the top 15 important OTUs). Random Forest classification misclassified 6 of the 15 samples when comparing pasteurized *vs* non‐pasteurized samples, and misclassified 1 of the Gadut_producer_1 samples as coming from Padang Panjang (data not shown).

Previous studies focusing on potential probiotics isolated from dadih primarily isolated lactobacilli and enterococci (Collado *et al*. 2007) rather than lactococci. These studies used conventional culture‐dependent techniques, with plating on MRS agar, which does not allow for growth of *Lactococcus*. The current next‐generation sequencing approach determined the major microbes present in dadih, and showed that lactobacilli and enterococci are actually present in minor proportions. It suggests reconsidering whether other microbes are (also) important for the health beneficial properties of the fermented buffalo milk (Surono 2003; Collado *et al*. 2007; Surono *et al*. 2011; Kusuma *et al*. 2015).

In conclusion, fermentation results differ significantly from producer to producer and based on the presence of *Klebsiella* and *Enterobacteriaceae* back‐slopping practice would be advisable. However, even with back‐slopping *Klebsiella* as a marker for poor hygienic conditions and *Enterobacteriaceae* as a potential pathogen are not completely suppressed and therefore, in terms of potential food safety, more research is required to follow the complete fermentation process, and to decipher the origin of these microbes, despite the lack of food poisoning reports. Global consumption of the product requires the establishment of good manufacturing practices.

## Materials and methods

### Source of dadih samples

Dadih samples were collected from two different regions in Bukittinggi city (Gadut (2 separate producers) and Palupuh), and in Padang Panjang town. These regions were chosen as the ‘hot‐spots’ of dadih production in Indonesia. Two samples from each producer were obtained: the raw buffalo milk used for fermentation was either pasteurized (four samples) or not (four samples). Morever, there were differences in back‐slopping practice, where previous fermentations were used as a starter culture (five samples) or not (three samples). Table 1 gives an overview of the different samples obtained. Duplicate samples were stored frozen before nucleic acid extraction.

### Extraction of nucleic acid

The samples were centrifuged at 14 500***g*** (Centurion K243R, BRK5424 Microtube Rotor, Elscolab, Terschuur, the Netherlands), and subsequent DNA extraction from the pellet was performed using the Quick‐DNA™ Fecal/Soil Microbe Miniprep Kit (Zymo Research, Irvine, CA) according to manufacturer's instructions, using the Precellys 24 tissue homogenizer (Bertin Instruments, Montigny‐le‐Bretonneux, France), applying three cycles of 30 s each, with 5 min cooling on ice in between.

### PCR‐amplifying the V3–V4 region of the 16S rRNA gene and next generation sequencing

Illumina 16S rRNA gene amplicon libraries were generated and sequenced at BaseClear (Leiden, the Netherlands). In short, barcoded amplicons from the V3–V4 region of 16S rRNA genes were generated using a 2‐step PCR. 10–25 ng isolated genomic DNA was used as template for the first PCR with a total volume of 50 μl using the 341F (5’‐CCTACGGGNGGCWGCAG‐3’) and the 785R (5’‐GACTACHVGGGTATCTAATCC‐3’) primers appended with Illumina adaptor sequences (Illumina, San Diego, CA). PCR products were purified and the size of the PCR products were checked on Fragment analyzer (Advanced Analytical Technologies, Heidelberg, Germany) and quantified by fluorometric analysis. Purified PCR products were used for the 2nd PCR in combination with sample‐specific barcoded primers (Nextera XT index kit, Illumina). Subsequently, PCR products were purified, checked on a Fragment analyzer (Advanced Analytical Technologies) and quantified, followed by multiplexing, clustering and sequencing on an Illumina MiSeq with the paired‐end (2×) 300 bp protocol and indexing.

### Sequence processing and analyses

The sequencing run was analysed with the Illumina CASAVA pipeline (v1.8.3) with demultiplexing based on sample‐specific barcodes. The raw sequencing data produced was processed removing the sequence reads of too low quality (only ‘passing filter’ reads were selected) and discarding reads containing adaptor sequences or PhiX control with an in‐house filtering protocol. A quality assessment on the remaining reads was performed using the FASTQC quality control tool ver. 0.10.0. Subsequently, the sequences were further analysed using the Quantitative Insights Into Microbial Ecology (QIIME) software pipeline, ver. 1.9.1 (Caporaso *et al*. 2010) for α‐ and β‐diversity and (un)weighted principal coordinate analysis. Linear discriminant analysis effect size (LEfSe) (Segata *et al*. 2011) was used to find biomarkers between groups using relative abundances from the OTU tables generated in QIIME. Multivariate statistical analyses on the data were done using metagenassist (Arndt *et al*. 2012).

## Conflict of Interest

Authors declare no conflict of interest.

## Supporting information


**Figure S1** (A) Weighted UniFrac of the dadih samples, colored according to starter culture use (back slopping; 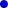) or not (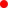). Orientation of the graph is the same as in Figure 2A. (B) Weighted UniFrac of the dadih samples, colored according to use of pasteurized buffalo milk (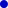) or not (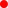). Orientation of the graph is the same as in Figure 2A.
**Figure S2** Correlation between the presence (abundance %) of *Klebsiella* and the presence of another *Enterobacteriaceae* OTU. The line displays the linear trendline.
**Figure S3** Co‐occurence of the different OTUs in the samples.
**Figure S4** Random Forest classification. Top 15 OTUs used for correct classification of samples in the category ‘starter culture use’ or ‘no starter culture use’. Insert: classification table.
**Table S1** Relative abundance of the different OTUs in the duplicate dadih samples. Shaded rows are indicated in Figure 1.
