# Supplementary material for: Microbiota composition of dadih – a traditional fermented buffalo milk of West Sumatra
Source: Lett Appl Microbiol. 2019 Jan 31;68(3):234–40. doi: 10.1111/lam.13107 (PMC6849839; doi:10.1111/lam.13107)
Supplement: Supplementary file 1 — Figure S1 (A) Weighted UniFrac of the dadih samples, colored according to starter culture use (back slopping; ) or not (). Orientation of the graph is the same as in Figure 2A. (B) Weighted UniFrac of the dadih samples, colored according to use of pasteurized buffalo milk () or not (). Orientation of the graph is the same as in Figure 2A. Figure S2 Correlation between the presence (abundance %) of Klebsiella and the presence of another Enterobacteriaceae OTU. The line displays the linear trendline. Figure S3 Co‐occurence of the different OTUs in the samples. Figure S4 Random Forest classification. Top 15 OTUs used for correct classification of samples in the category ‘starter culture use’ or ‘no starter culture use’. Insert: classification table. Table S1 Relative abundance of the different OTUs in the duplicate dadih samples. Shaded rows are indicated in Figure 1. [file LAM-68-234-s001.docx]

Supplementary Figure 1A.


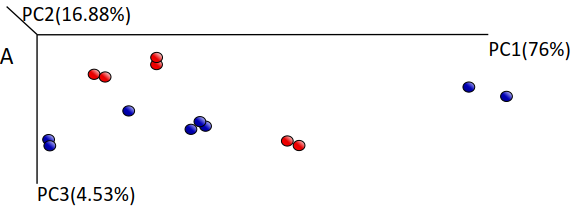


Suppl Figure 1A. Weighted UniFrac of the dadih samples, colored according to starter culture use (back slopping; ⏺) or not (⏺). Orientation of the graph is the same as in Figure 2A.

Supplementary Figure 1B.


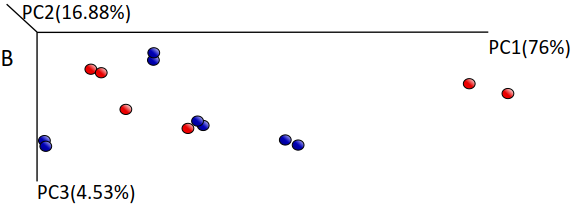


Suppl Figure 1B. Weighted UniFrac of the dadih samples, colored according to use of pasteurized buffalo milk (⏺) or not (⏺).Orientation of the graph is the same as in Figure 2A.

Supplementary Figure S2.

Suppl Figure 2. Correlation between presence (abundance %) of *Klebsiella* and presence of another *Enterobacteriaceae* OTU. The line displays the linear trendline.

Supplementary Figure 3.


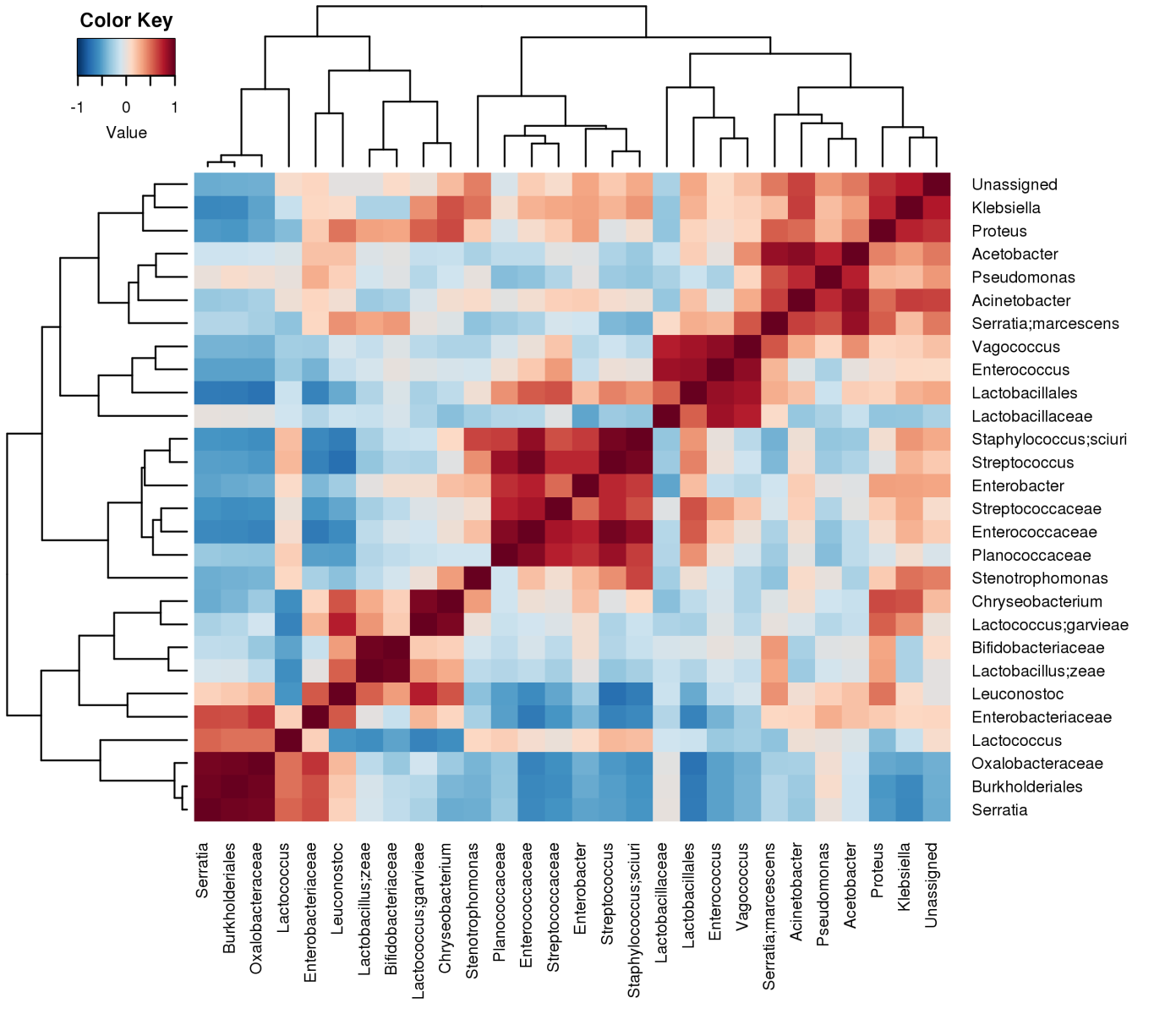


Suppl Figure 3. Co-occurence of the different OTUs in the samples.

Supplementary Figure 4.

Suppl Figure 4. Random Forest classification. Top 15 OTUs used for correct classification of samples in the category ‘starter culture use’ or ‘no starter culture use’. Insert: classification table.

Supplementary Table 1. Relative abundance of the different OTUs in the duplicate dadih samples. Shaded rows are indicated in Figure 1.

| #OTU ID | A1 | A2 | B2 | C1 | C2 | D1 | D2 | E1 | E2 | F1 | F2 | G1 | G2 | H1 | H2 |
| --- | --- | --- | --- | --- | --- | --- | --- | --- | --- | --- | --- | --- | --- | --- | --- |
| Unassigned | 1.83% | 2.14% | 1.46% | 1.82% | 1.43% | 1.82% | 1.57% | 1.45% | 1.31% | 1.94% | 1.66% | 0.90% | 0.66% | 1.44% | 1.30% |
| g__Brevibacterium | 0.00% | 0.00% | 0.00% | 0.10% | 0.08% | 0.09% | 0.11% | 0.00% | 0.00% | 0.00% | 0.00% | 0.00% | 0.00% | 0.00% | 0.00% |
| g__Corynebacterium | 0.06% | 0.06% | 0.00% | 0.12% | 0.14% | 0.38% | 0.41% | 0.00% | 0.00% | 0.00% | 0.00% | 0.00% | 0.00% | 0.00% | 0.00% |
| f__Dermabacteraceae;g__ | 0.00% | 0.00% | 0.00% | 0.00% | 0.01% | 0.01% | 0.01% | 0.00% | 0.00% | 0.00% | 0.00% | 0.00% | 0.00% | 0.00% | 0.00% |
| g__Brachybacterium | 0.00% | 0.00% | 0.00% | 0.00% | 0.00% | 0.00% | 0.00% | 0.00% | 0.01% | 0.00% | 0.00% | 0.00% | 0.00% | 0.00% | 0.00% |
| g__Leucobacter | 0.00% | 0.00% | 0.00% | 0.00% | 0.01% | 0.01% | 0.01% | 0.00% | 0.00% | 0.00% | 0.00% | 0.00% | 0.00% | 0.00% | 0.00% |
| g__Microbacterium | 0.00% | 0.00% | 0.00% | 0.00% | 0.00% | 0.00% | 0.00% | 0.00% | 0.00% | 0.00% | 0.00% | 0.00% | 0.00% | 0.00% | 0.00% |
| f__Micrococcaceae;g__ | 0.00% | 0.00% | 0.00% | 0.09% | 0.13% | 0.16% | 0.17% | 0.00% | 0.00% | 0.00% | 0.00% | 0.00% | 0.00% | 0.00% | 0.00% |
| g__Kocuria | 0.00% | 0.00% | 0.00% | 0.09% | 0.08% | 0.07% | 0.07% | 0.00% | 0.00% | 0.00% | 0.00% | 0.00% | 0.00% | 0.00% | 0.00% |
| f__Nocardiopsaceae | 0.00% | 0.00% | 0.00% | 0.00% | 0.00% | 0.00% | 0.00% | 0.00% | 0.00% | 0.00% | 0.00% | 0.00% | 0.00% | 0.00% | 0.00% |
| g__Nocardiopsis | 0.00% | 0.00% | 0.00% | 0.00% | 0.00% | 0.00% | 0.00% | 0.04% | 0.04% | 0.00% | 0.00% | 0.00% | 0.00% | 0.00% | 0.00% |
| g__Prauseria | 0.00% | 0.00% | 0.00% | 0.00% | 0.00% | 0.00% | 0.00% | 0.01% | 0.00% | 0.00% | 0.00% | 0.00% | 0.00% | 0.00% | 0.00% |
| g__Streptomyces | 0.00% | 0.00% | 0.00% | 0.00% | 0.00% | 0.00% | 0.00% | 0.02% | 0.01% | 0.00% | 0.00% | 0.00% | 0.00% | 0.00% | 0.00% |
| f__Bifidobacteriaceae;g__ | 0.13% | 0.14% | 1.21% | 17.78% | 19.31% | 0.39% | 0.44% | 0.08% | 0.09% | 1.30% | 1.46% | 0.00% | 0.00% | 0.01% | 0.00% |
| f__Coriobacteriaceae;g__ | 0.00% | 0.00% | 0.00% | 0.02% | 0.00% | 0.00% | 0.00% | 0.00% | 0.00% | 0.00% | 0.00% | 0.00% | 0.00% | 0.00% | 0.00% |
| g__Flavobacterium | 0.00% | 0.00% | 0.00% | 0.00% | 0.00% | 0.00% | 0.00% | 0.00% | 0.00% | 0.00% | 0.00% | 0.00% | 0.00% | 0.00% | 0.00% |
| g__Myroides | 0.01% | 0.01% | 0.00% | 0.00% | 0.00% | 0.00% | 0.00% | 0.00% | 0.00% | 0.00% | 0.00% | 0.00% | 0.00% | 0.00% | 0.00% |
| g__Chryseobacterium | 0.01% | 0.02% | 0.00% | 0.07% | 0.07% | 0.20% | 0.18% | 0.04% | 0.03% | 0.05% | 0.07% | 0.00% | 0.00% | 0.00% | 0.00% |
| g__Sphingobacterium | 0.00% | 0.00% | 0.00% | 0.00% | 0.01% | 0.01% | 0.01% | 0.00% | 0.00% | 0.00% | 0.00% | 0.00% | 0.00% | 0.00% | 0.00% |
| f__Bacillaceae;g__ | 0.05% | 0.04% | 0.09% | 0.00% | 0.00% | 0.00% | 0.00% | 0.00% | 0.00% | 0.00% | 0.00% | 0.00% | 0.00% | 0.00% | 0.00% |
| f__Planococcaceae;g__ | 0.01% | 0.00% | 0.00% | 0.01% | 0.00% | 0.00% | 0.00% | 0.09% | 0.08% | 0.00% | 0.00% | 0.00% | 0.00% | 0.00% | 0.01% |
| g__Macrococcus | 0.02% | 0.01% | 0.00% | 0.00% | 0.00% | 0.00% | 0.00% | 0.00% | 0.00% | 0.00% | 0.00% | 0.00% | 0.00% | 0.00% | 0.00% |
| g__Staphylococcus | 0.00% | 0.01% | 0.00% | 0.02% | 0.02% | 0.02% | 0.04% | 0.20% | 0.17% | 0.18% | 0.12% | 0.00% | 0.00% | 0.00% | 0.00% |
| o__Lactobacillales | 0.00% | 0.00% | 0.00% | 0.00% | 0.00% | 0.00% | 0.00% | 0.00% | 0.00% | 0.00% | 0.00% | 0.00% | 0.00% | 0.00% | 0.00% |
| o__Lactobacillales;f__;g__ | 0.15% | 0.18% | 0.43% | 0.10% | 0.08% | 0.05% | 0.05% | 0.23% | 0.22% | 0.13% | 0.10% | 0.01% | 0.01% | 0.00% | 0.01% |
| f__Enterococcaceae | 0.07% | 0.08% | 0.11% | 0.10% | 0.11% | 0.07% | 0.07% | 0.54% | 0.47% | 0.19% | 0.17% | 0.00% | 0.01% | 0.00% | 0.01% |
| f__Enterococcaceae;g__ | 0.00% | 0.00% | 0.00% | 0.00% | 0.00% | 0.00% | 0.00% | 0.00% | 0.00% | 0.00% | 0.00% | 0.00% | 0.00% | 0.00% | 0.00% |
| g__Enterococcus | 0.02% | 0.03% | 0.15% | 0.02% | 0.02% | 0.03% | 0.02% | 0.02% | 0.04% | 0.02% | 0.01% | 0.00% | 0.00% | 0.00% | 0.00% |
| g__Vagococcus | 0.48% | 0.52% | 1.25% | 0.14% | 0.14% | 0.07% | 0.10% | 0.16% | 0.11% | 0.04% | 0.09% | 0.00% | 0.00% | 0.00% | 0.00% |
| f__Lactobacillaceae | 0.48% | 0.50% | 12.04% | 0.08% | 0.11% | 0.02% | 0.01% | 0.00% | 0.00% | 0.00% | 0.00% | 1.68% | 2.08% | 0.93% | 0.94% |
| f__Lactobacillaceae;g__ | 0.24% | 0.27% | 6.19% | 0.80% | 0.98% | 0.18% | 0.27% | 0.00% | 0.00% | 0.00% | 0.00% | 1.04% | 1.42% | 0.63% | 0.73% |
| g__Lactobacillus | 0.01% | 0.01% | 0.06% | 4.59% | 5.83% | 1.19% | 1.22% | 0.00% | 0.00% | 0.00% | 0.00% | 0.38% | 0.46% | 0.16% | 0.13% |
| g__Pediococcus | 0.00% | 0.00% | 0.02% | 0.00% | 0.00% | 0.00% | 0.00% | 0.00% | 0.00% | 0.00% | 0.00% | 0.00% | 0.00% | 0.00% | 0.00% |
| f__Leuconostocaceae;g__ | 0.00% | 0.00% | 0.00% | 0.03% | 0.03% | 0.01% | 0.00% | 0.00% | 0.00% | 0.00% | 0.00% | 0.00% | 0.00% | 0.00% | 0.00% |
| g__Leuconostoc | 2.85% | 2.86% | 1.34% | 4.40% | 4.92% | 6.47% | 6.11% | 0.00% | 0.00% | 0.00% | 0.00% | 2.24% | 3.04% | 1.96% | 2.06% |
| g__Weissella | 0.00% | 0.00% | 0.00% | 0.01% | 0.00% | 0.00% | 0.00% | 0.00% | 0.00% | 0.00% | 0.00% | 0.00% | 0.00% | 0.00% | 0.00% |
| f__Streptococcaceae | 0.01% | 0.00% | 0.01% | 0.00% | 0.00% | 0.00% | 0.01% | 0.02% | 0.04% | 0.01% | 0.01% | 0.00% | 0.00% | 0.00% | 0.00% |
| f__Streptococcaceae;g__ | 0.00% | 0.00% | 0.00% | 0.00% | 0.00% | 0.00% | 0.00% | 0.00% | 0.00% | 0.00% | 0.00% | 0.00% | 0.00% | 0.00% | 0.00% |
| g__Lactococcus | 65.02% | 65.64% | 60.18% | 53.30% | 51.17% | 55.44% | 57.84% | 69.65% | 70.04% | 67.68% | 68.71% | 83.66% | 83.00% | 76.20% | 76.17% |
| g__Streptococcus | 0.62% | 0.57% | 0.01% | 0.02% | 0.00% | 0.01% | 0.00% | 9.31% | 7.88% | 4.66% | 4.28% | 0.00% | 0.02% | 0.00% | 0.00% |
| o__Clostridiales;f__;g__ | 0.00% | 0.00% | 0.00% | 0.01% | 0.00% | 0.00% | 0.00% | 0.00% | 0.00% | 0.00% | 0.00% | 0.00% | 0.00% | 0.00% | 0.00% |
| f__Clostridiaceae;g__ | 0.00% | 0.00% | 0.00% | 0.00% | 0.00% | 0.00% | 0.00% | 0.00% | 0.00% | 0.00% | 0.00% | 0.00% | 0.00% | 0.00% | 0.00% |
| g__Clostridium | 0.00% | 0.00% | 0.00% | 0.00% | 0.00% | 0.00% | 0.00% | 0.00% | 0.00% | 0.00% | 0.00% | 0.00% | 0.00% | 0.00% | 0.00% |
| f__Lachnospiraceae;g__ | 0.00% | 0.00% | 0.00% | 0.01% | 0.00% | 0.00% | 0.00% | 0.00% | 0.00% | 0.00% | 0.00% | 0.00% | 0.00% | 0.00% | 0.00% |
| g__Succiniclasticum | 0.00% | 0.00% | 0.00% | 0.01% | 0.00% | 0.00% | 0.00% | 0.00% | 0.00% | 0.00% | 0.00% | 0.00% | 0.00% | 0.00% | 0.00% |
| f__Brucellaceae;g__ | 0.00% | 0.00% | 0.00% | 0.00% | 0.00% | 0.00% | 0.01% | 0.00% | 0.00% | 0.00% | 0.00% | 0.00% | 0.00% | 0.00% | 0.00% |
| g__Ochrobactrum | 0.00% | 0.00% | 0.00% | 0.00% | 0.01% | 0.00% | 0.00% | 0.00% | 0.00% | 0.00% | 0.00% | 0.00% | 0.00% | 0.00% | 0.00% |
| g__Methylosinus | 0.00% | 0.00% | 0.00% | 0.00% | 0.00% | 0.00% | 0.00% | 0.00% | 0.00% | 0.00% | 0.00% | 0.00% | 0.00% | 0.00% | 0.00% |
| g__Agrobacterium | 0.00% | 0.00% | 0.00% | 0.00% | 0.00% | 0.00% | 0.00% | 0.00% | 0.00% | 0.00% | 0.00% | 0.00% | 0.00% | 0.00% | 0.00% |
| g__Acetobacter | 1.00% | 1.00% | 0.00% | 0.14% | 0.24% | 0.08% | 0.06% | 0.00% | 0.00% | 0.00% | 0.00% | 0.02% | 0.01% | 0.02% | 0.02% |
| g__Gluconobacter | 0.00% | 0.00% | 0.00% | 0.00% | 0.01% | 0.00% | 0.00% | 0.00% | 0.00% | 0.00% | 0.00% | 0.00% | 0.00% | 0.00% | 0.00% |
| c__Betaproteobacteria | 0.00% | 0.00% | 0.00% | 0.00% | 0.00% | 0.00% | 0.00% | 0.00% | 0.00% | 0.00% | 0.00% | 0.00% | 0.00% | 0.00% | 0.00% |
| o__Burkholderiales | 0.00% | 0.00% | 0.00% | 0.00% | 0.00% | 0.00% | 0.00% | 0.00% | 0.00% | 0.00% | 0.00% | 0.01% | 0.01% | 0.01% | 0.01% |
| f__Alcaligenaceae | 0.00% | 0.00% | 0.00% | 0.00% | 0.00% | 0.00% | 0.00% | 0.00% | 0.00% | 0.00% | 0.00% | 0.00% | 0.00% | 0.00% | 0.00% |
| g__Achromobacter | 0.00% | 0.00% | 0.00% | 0.01% | 0.00% | 0.02% | 0.01% | 0.00% | 0.00% | 0.00% | 0.00% | 0.00% | 0.00% | 0.00% | 0.00% |
| f__Comamonadaceae;g__ | 0.00% | 0.00% | 0.00% | 0.00% | 0.00% | 0.00% | 0.00% | 0.00% | 0.00% | 0.00% | 0.00% | 0.00% | 0.00% | 0.00% | 0.00% |
| f__Oxalobacteraceae;g__ | 0.18% | 0.20% | 0.01% | 0.08% | 0.07% | 0.34% | 0.27% | 0.00% | 0.00% | 0.00% | 0.00% | 0.75% | 0.71% | 0.72% | 0.60% |
| c__Gammaproteobacteria | 0.00% | 0.00% | 0.00% | 0.00% | 0.00% | 0.00% | 0.00% | 0.00% | 0.00% | 0.00% | 0.00% | 0.00% | 0.00% | 0.00% | 0.00% |
| f__Aeromonadaceae;g__ | 0.00% | 0.00% | 0.00% | 0.00% | 0.00% | 0.00% | 0.00% | 0.00% | 0.00% | 0.00% | 0.00% | 0.00% | 0.01% | 0.00% | 0.00% |
| g__Shewanella | 0.00% | 0.00% | 0.00% | 0.00% | 0.00% | 0.00% | 0.00% | 0.00% | 0.00% | 0.00% | 0.00% | 0.00% | 0.00% | 0.00% | 0.00% |
| f__Enterobacteriaceae | 2.37% | 2.16% | 1.14% | 2.12% | 1.94% | 2.65% | 2.69% | 0.35% | 0.46% | 0.94% | 0.95% | 0.89% | 0.89% | 1.53% | 1.70% |
| f__Enterobacteriaceae;g__ | 1.93% | 1.74% | 0.37% | 1.04% | 0.92% | 2.94% | 2.69% | 0.48% | 0.67% | 0.94% | 0.83% | 2.40% | 2.30% | 4.61% | 4.29% |
| g__Citrobacter | 0.01% | 0.00% | 0.00% | 0.00% | 0.00% | 0.01% | 0.00% | 0.00% | 0.00% | 0.00% | 0.00% | 0.00% | 0.00% | 0.00% | 0.00% |
| g__Enterobacter | 0.01% | 0.00% | 0.00% | 0.01% | 0.00% | 0.01% | 0.00% | 0.02% | 0.01% | 0.01% | 0.01% | 0.00% | 0.00% | 0.00% | 0.00% |
| g__Klebsiella | 21.34% | 20.59% | 13.74% | 12.30% | 11.73% | 26.58% | 24.88% | 15.68% | 16.87% | 19.29% | 18.55% | 5.85% | 5.24% | 11.56% | 11.79% |
| g__Morganella | 0.01% | 0.00% | 0.00% | 0.03% | 0.04% | 0.00% | 0.00% | 0.00% | 0.00% | 0.00% | 0.00% | 0.00% | 0.00% | 0.00% | 0.00% |
| g__Plesiomonas | 0.00% | 0.00% | 0.00% | 0.00% | 0.00% | 0.00% | 0.00% | 0.00% | 0.00% | 0.00% | 0.00% | 0.01% | 0.01% | 0.01% | 0.02% |
| g__Proteus | 0.23% | 0.18% | 0.11% | 0.24% | 0.16% | 0.22% | 0.27% | 0.12% | 0.14% | 0.15% | 0.14% | 0.07% | 0.05% | 0.08% | 0.10% |
| g__Providencia | 0.00% | 0.01% | 0.00% | 0.07% | 0.04% | 0.03% | 0.02% | 0.00% | 0.00% | 0.00% | 0.00% | 0.00% | 0.00% | 0.00% | 0.00% |
| g__Serratia | 0.10% | 0.13% | 0.06% | 0.09% | 0.08% | 0.01% | 0.04% | 0.00% | 0.00% | 0.00% | 0.00% | 0.04% | 0.04% | 0.04% | 0.05% |
| g__Legionella | 0.00% | 0.00% | 0.00% | 0.00% | 0.00% | 0.01% | 0.01% | 0.00% | 0.00% | 0.00% | 0.00% | 0.00% | 0.00% | 0.00% | 0.00% |
| g__Halomonas | 0.00% | 0.00% | 0.00% | 0.00% | 0.00% | 0.00% | 0.00% | 0.00% | 0.00% | 0.00% | 0.00% | 0.00% | 0.00% | 0.00% | 0.00% |
| f__Moraxellaceae | 0.00% | 0.00% | 0.00% | 0.00% | 0.00% | 0.00% | 0.00% | 0.00% | 0.00% | 0.00% | 0.00% | 0.00% | 0.00% | 0.00% | 0.00% |
| f__Moraxellaceae;g__ | 0.00% | 0.01% | 0.00% | 0.00% | 0.00% | 0.00% | 0.00% | 0.00% | 0.00% | 0.00% | 0.00% | 0.00% | 0.00% | 0.00% | 0.00% |
| g__Acinetobacter | 0.70% | 0.83% | 0.00% | 0.05% | 0.05% | 0.19% | 0.20% | 1.44% | 1.24% | 2.16% | 2.53% | 0.02% | 0.02% | 0.05% | 0.06% |
| g__Enhydrobacter | 0.00% | 0.00% | 0.00% | 0.00% | 0.00% | 0.00% | 0.00% | 0.00% | 0.00% | 0.00% | 0.00% | 0.00% | 0.00% | 0.00% | 0.00% |
| g__Pseudomonas | 0.01% | 0.02% | 0.00% | 0.01% | 0.00% | 0.00% | 0.00% | 0.00% | 0.00% | 0.00% | 0.01% | 0.00% | 0.01% | 0.01% | 0.00% |
| f__Xanthomonadaceae | 0.00% | 0.00% | 0.00% | 0.04% | 0.03% | 0.07% | 0.05% | 0.00% | 0.00% | 0.00% | 0.00% | 0.00% | 0.00% | 0.00% | 0.00% |
| g__Stenotrophomonas | 0.00% | 0.01% | 0.00% | 0.02% | 0.01% | 0.12% | 0.08% | 0.03% | 0.03% | 0.30% | 0.30% | 0.00% | 0.00% | 0.00% | 0.00% |
